# The Relationship of Allergy Severity to Depressive and Anxious Symptomatology: The Role of Attitude toward Illness

**DOI:** 10.5402/2011/765309

**Published:** 2012-01-12

**Authors:** Elizabeth S. Molzon, Kristina I. Suorsa, Stephanie E. Hullmann, Jamie L. Ryan, Larry L. Mullins

**Affiliations:** Department of Psychology, Oklahoma State University, Stillwater, OK 74078, USA

## Abstract

The current study examined the relationship between self-reported allergy severity, depressive and anxious symptoms, and attitude toward illness in adolescents and young adults (AYAs) with allergies. Participants were 214 undergraduate students between the ages of 17–25 years with self-reported allergies. Participants completed the Center for Epidemiological Studies Depression Scale (CES-D), the Zung Self-Rating Anxiety Scale (SAS), and the Child Attitude Toward Illness Scale (CATIS) as measures of depressive symptoms, anxious symptoms, and attitude toward illness, respectively. Using the bootstrapping method, results revealed that attitude toward illness mediated the relationship between self-reported disease severity and depressive and anxious symptoms. Results of the current study suggest that attitude toward illness is one pathway by which subjective disease severity impacts psychological functioning in AYAs with allergies.

## 1. Introduction

Allergies represent one of the most prevalent chronic illnesses in the United States, affecting 40–50 million individuals [1]. Although allergies are often viewed as non-life-threatening, they are increasingly recognized to have a significant negative impact on one's social, emotional, occupational, and physical functioning [2, 3].

Much of the research regarding psychosocial adjustment to allergies focuses primarily on younger children and on specific subgroups of allergies (i.e., food-related allergies), rather than the broader disease itself [4]. Examinations of adults with allergies have demonstrated that adolescents and young adults (AYAs) experience pejorative psychosocial outcomes compared to healthy peers [5, 6]. Relatively few studies have examined the impact of allergy symptom severity on these psychosocial outcomes; however, Kovács and colleagues [7] found that AYAs and adults who reported more depressive symptoms also reported more severe allergic complaints. Additionally, for AYAs with asthma, greater asthma symptom severity was found to be significantly related to increased depressive and anxious symptoms [8]. What has yet to be identified, however, are factors that potentially mediate or explain the precise relationship between allergy severity and negative psychosocial outcomes.

Importantly, researchers have demonstrated a strong relationship between attitude toward illness and anxious and depressive symptoms. LeBovidge and colleagues [9] found that children with arthritis who have a positive attitude toward their illness have lower levels of depressive and anxious symptoms. A similar relationship has been demonstrated in children with food allergies, such that a more negative attitude toward their allergies was associated with increased symptoms of anxiety and depression [10]. To our knowledge, no researchers have examined the impact of attitude toward illness on psychosocial outcomes in AYAs with allergies. It may be that AYAs who view their illness negatively (i.e., evidence more negative attitudes) are more likely to withdraw from others and allow their illness to impact their daily activities [9, 11]. In contrast, those who have a more positive attitude toward their illness may be more resilient and open to new opportunities [11].

To understand how illness attitudes may influence the relationship between illness severity and psychosocial functioning, Lazarus and Folkman's stress, appraisal, and coping model was utilized [12] in the current study. This model suggests that the way an individual appraises or thinks about a stressor will predict how well he/she copes with that stressor. In the context of AYAs with allergies, allergies serve as the stressor with which an individual must cope. Greater allergy symptom severity may negatively impact an individual's attitude toward his/her illness because symptoms interfere with daily tasks and, in turn, cause distress. From the extant research on attitude toward illness, we know that a young adult's attitude toward his/her illness ostensibly determines psychological adjustment, including depressive and anxious symptomatology. Using this model as a theoretical framework, it was hypothesized that attitude toward illness would mediate the relationship between allergy severity and depressive or anxious symptoms, such that, attitude toward illness would clarify and explain part of the relationship between allergy severity and depressive or anxious symptoms.

## 2. Methods

### 2.1. Participants

The current study included 214 undergraduate students between the ages of 17–25 years (*M* = 19.52, SD = 1.50) with self-reported allergies. The majority of the AYAs were female (73.4%) and most self-identified as Caucasian (80.4%). The ethnic breakdown is consistent with the ethnic distribution of the university where the study was conducted; demographic information can be found in Table 1. Participants reported whether they experienced allergic reactions to environmental, food, animal, or other allergens. Information regarding allergy severity and number of allergens are reported in Table 2.

### 2.2. Measures

#### 2.2.1. Demographic Questionnaire

Participants completed a demographic questionnaire where they provided information regarding their age, sex, ethnicity, education level. They also provided information about their allergies, including self-reported disease severity on a 5-point Likert scale and types of allergies.

#### 2.2.2. Center for Epidemiological Students Depression Scale (CES-D)

The CES-D is a 20-item, self-report measure of depressive symptoms [13]. For each item, participants indicated whether they had experienced each symptom within the last week. Each of the items is ranked on a four-point Likert scale, ranging from occurring *rarely or none of the time* to *most or all of the time*. Responses were summed to calculate a total score, with greater scores representing higher levels of depressive symptomatology. Scores above 16 indicate clinically significant levels of depression [13]. Good to excellent (.85–.90) internal consistency reliability has been demonstrated for the CES-D [13]. Cronbach's alpha for the CES-D total score for the current sample was excellent at  .90.

#### 2.2.3. Zung Self-Rating Anxiety Scale (SAS)

The SAS is a 20-item, self-report measure of anxious symptoms [14]. For each item, participants indicated the extent to which they had experienced each symptom within the last week. Each of the items is ranked on a four-point Likert scale, ranging from occurring *none or a little of the time* to *most of the time*. Responses were summed to calculate a total score, with higher scores indicating greater levels of anxious symptomatology. Scores above 45 suggest clinically significant levels of anxiety [14]. Good (.71) internal consistency reliability has been demonstrated for the SAS [14]. Cronbach's alpha for the SAS total score for the current sample was good at  .86.

#### 2.2.4. Child Attitude toward Illness Scale (CATIS)

The CATIS is a 13-item, self-report measure of an individual's feelings about having a chronic illness [11]. Each item is ranked on a five-point Likert scale, ranging from *very bad or never* to *very good or very often*. A mean response for all items is then calculated, with higher scores indicating more positive illness attitudes. Scores on the CATIS range from 1 to 5. The CATIS was first validated with children aged 8–12 years with epilepsy and asthma [11] and has been adapted for individuals with other chronic illnesses, including type 1 diabetes [15], juvenile rheumatic diseases [16], and allergies [10]. The CATIS has also been validated in a sample of adolescents with epilepsy [17]. For the current study, the CATIS was adapted from the original version for individuals with allergies by changing items to include “allergies” (e.g., *how often do you feel different from others because of your allergies?*). Good (.87–.89) internal consistency reliability has been demonstrated for the CATIS in an adolescent population [17]. Cronbach's alpha for the CATIS total score for the current sample was good at  .82.

### 2.3. Procedure

Participants were recruited from an online participant pool for undergraduate students at a large midwestern university. The participants self-identified having allergies and completed all measures online as part of a larger study examining psychosocial adjustment in college students with allergies. The study had approval from the university's Institutional Review Board, and all participants received research credit for an undergraduate class in exchange for their participation.

## 3. Results

### 3.1. Preliminary Analyses

A series of bivariate correlations was first conducted to determine if any demographic variables (i.e., age, gender, and ethnicity) were related to any outcome variables (i.e., CES-D total score, SAS total score, and CATIS total score). Results revealed that gender was significantly related to CES-D total score, *r* = .15, *P* = .02, SAS total score, *r* = .27, *P* < .001, and CATIS total score, *r* = −.21, *P* = .002. Specifically, female AYAs reported higher levels of depressive and anxious symptoms and had more negative attitudes toward their illness than male AYAs. No other significant relationships were identified. Accordingly, gender was entered as a covariate in all regression analyses. The ranges, means, and standard deviations for the outcome variables can be found in Table 3. The sample was also examined to determine the number of AYAs reporting clinically significant levels of depressive and anxious symptoms. Using the clinical cutoff scores identified by the authors [13, 14], 43.5% of the sample were found to be experiencing clinically significant levels of depressive symptoms, and 22.4% were found to be experiencing clinically significant levels of anxious symptoms. This information is also available in Table 3.

### 3.2. Primary Analyses

Analyses of the direct and indirect effects of allergy severity on anxious and depressive symptoms were conducted using the recommendations of Preacher and Hayes [18, 19], who recommend using bootstrapping to decrease Type 1 error rates and increase power. The indirect macro developed for SPSS by Preacher and Hayes [18] was utilized in the following analyses. The data was resampled 5000 times.

 For the depressive symptoms model, a significant direct relationship was observed between subjective allergy symptom severity and CATIS total score, *t*(213) = −3.59, *P* < .001, indicating that AYAs who reported more severe symptoms had more negative attitudes toward their illness. Another significant direct relationship was observed between CATIS and CES-D total scores, *t*(213) = −6.44, *P* < .001, such that AYAs with more negative attitudes toward their illness reported more depressive symptoms. A nonsignificant direct relationship was observed between disease severity and CES-D total score, *t*(213) = 1.70, *P* = .09. However, after controlling for CATIS total scores, the direct relationship between subjective allergy severity and depressive symptoms became less significant, and the overall model was significant, *F*(3, 210) = 17.03, *P* < .001. The partial effect of gender was not significant, suggesting that gender did not impact the relationship between disease severity and depressive symptoms. As zero did not fall within the 95% confidence interval, post hoc bootstrapping results supported the mediation results (95%  CI = .56 to 2.34).

 For the anxious symptoms model, a significant direct relationship was observed between subjective allergy symptom severity and CATIS total score, *t*(213) = −3.59, *P* < .001, indicating that AYAs who reported more severe symptoms had more negative illness attitudes. Another significant direct relationship was observed between CATIS and SAS total scores, *t*(213) = −6.70, *P* < .001, such that AYAs with more negative attitudes toward their illness reported more anxious symptoms. Also, a significant direct relationship was observed between subjective allergy symptom severity and SAS total score, *t*(213) = 2.47, *P* = .01, such that individuals with more severe allergy symptoms reported more anxious symptoms. After controlling for CATIS total scores, the relationship between allergy severity and anxious symptoms was no longer significant, thus confirming that illness attitudes mediated the relation between allergy severity and anxious symptoms, *F*(3,210) = 24.36, *P* < .001. The partial effect of gender was significant, *t*(213) = 2.77, *P* = .006, suggesting that gender may impact the relationship between disease severity and anxious symptoms; however, as zero did not fall within the 95% confidence interval, post hoc bootstrapping results supported the mediation results (95%  CI = .52  to  2.23).

## 4. Discussion

The results of the current investigation suggest that allergy symptom severity is significantly related to anxious symptoms and that attitude toward illness significantly mediates the relationship between allergy severity and both depressive and anxious symptoms in AYAs, after controlling for gender. These results indeed suggest that illness attitudes can significantly impact psychological functioning in AYAs with allergies.

In contrast with previous research [7], we did not find that self-reported disease severity was significantly related to depressive symptoms, perhaps due to the exclusion of individuals with comorbid asthma in the current study. However, there was a significant relationship between self-reported allergy severity and anxious symptoms, such that the AYAs with more severe allergy symptoms reported more anxious symptoms. For both models, attitude toward illness significantly mediated the relationship between disease severity and depressive and anxious symptoms. The results of this study confirm that attitudes impact an individual's psychosocial functioning and reported disease severity, and these results are consistent with Lazarus and Folkman's stress, appraisal, and coping model, which posits that feelings/attitudes are indeed related to illness severity and experiences of distress [12]. Our results would indicate that attitude toward illness is one pathway in which disease severity impacts depressive and anxious symptoms in AYAs with allergies.

It is important to note that high rates of AYAs met the suggested clinical cutoffs based on their reported anxiety and depressive symptoms, as 43.5 and 22.4 percent met the cutoffs for depressive and anxiety symptoms, respectively. This suggests that although we utilized a nonclinical population, AYAs are indeed having significant adjustment difficulties. As such, our study suggests that the psychosocial adjustment of AYAs with allergies should be assessed periodically as they transition to adulthood.

There are several limitations to the current study. First, participants were recruited from a large midwestern university, and as a nonclinical population, they may not be representative of individuals with more severe symptoms. Participants also self-reported their diagnosis and severity levels, and this was not confirmed with physician report. Additionally, the psychosocial functioning was assessed using self-report measures, so the results may reflect shared method variance.

Overall, the current study builds upon past research examining the impact of severity on depressive and anxious symptoms and the role of attitude toward illness. The current study also adds to the literature examining psychosocial outcomes of AYAs with allergies. Such research is critical, as young adults remain an understudied population in chronic illness literature [20]. Clinically, our results would suggest that one's attitude toward their illness is a potential target for intervention to reduce depression and anxiety in AYAs with allergies.

## Figures and Tables

**Table 1:** Demographic characteristics.

Age (in years) *M* (SD)	19.52 (1.50)
Range	17–25
Gender (% female)	73.4
Ethnicity (%)	
Caucasian	80.4
African American	3.7
Hispanic	3.7
American Indian	7.5
Asian American	0.5
Multiracial	2.8
Other	1.4

**Table 2:** Illness parameters.

Subjective allergy severity (%)	
No reaction	3.3
Mild	26.8
Moderate	48.8
Severe	14.6
Extremely severe	4.9

Number of types of allergens (%)	
One allergen	44.9
Two allergens	39.3
Three allergens	12.1
Four allergens	3.7

Type of allergen (%)	
Environmental (pollen, dust, mold, etc.)	92.1
Animals (animal dander, insect bites, etc.)	37.4
Food (milk, eggs, peanuts, fish, etc.)	19.2
Other (latex, metals, drugs, etc.)	26.3

**Table 3:** Variables of interest.

	Observed Range	*M* (SD)	Number in clinical range (%)
CES-D	0–49	15.57 (10.15)	93 (43.5)
SAS	21–68	36.98 (9.45)	48 (22.4)
CATIS	2.08–4.77	3.39 (.52)	—

*Note*. CES-D = Center for Epidemiologic Studies Depression Scale clinical cutoff = 16; SAS = Zung Self-Rating Anxiety Scale, clinical cutoff = 45; CATIS = Child Attitude toward Illness Scale.
